# Ferroptosis: Final destination for cancer?

**DOI:** 10.1111/cpr.12761

**Published:** 2020-02-25

**Authors:** Zeng Ye, Wensheng Liu, Qifeng Zhuo, Qiangsheng Hu, Mengqi Liu, Qiqing Sun, Zheng Zhang, Guixiong Fan, Wenyan Xu, Shunrong Ji, Xianjun Yu, Yi Qin, Xiaowu Xu

**Affiliations:** ^1^ Department of Pancreatic Surgery Fudan University Shanghai Cancer Center Shanghai China; ^2^ Department of Oncology Shanghai Medical College Fudan University Shanghai China; ^3^ Shanghai Pancreatic Cancer Institute Shanghai China; ^4^ Pancreatic Cancer Institute Fudan University Shanghai China

**Keywords:** cancer therapy, ferroptosis, GPx4, lipid peroxidation

## Abstract

Ferroptosis is a recently defined, non‐apoptotic, regulated cell death (RCD) process that comprises abnormal metabolism of cellular lipid oxides catalysed by iron ions or iron‐containing enzymes. In this process, a variety of inducers destroy the cell redox balance and produce a large number of lipid peroxidation products, eventually triggering cell death. However, in terms of morphology, biochemistry and genetics, ferroptosis is quite different from apoptosis, necrosis, autophagy‐dependent cell death and other RCD processes. A growing number of studies suggest that the relationship between ferroptosis and cancer is extremely complicated and that ferroptosis promises to be a novel approach for the cancer treatment. This article primarily focuses on the mechanism of ferroptosis and discusses the potential application of ferroptosis in cancer therapy.

## 1 INTRODUCTION

Death is inevitable for any living cell, but death is also a complicated process. The Nomenclature Committee on Cell Death (NCCD) has formulated guidelines for defining cell death in view of morphology, biochemistry and function. In recent recommendations, they listed more than 12 types of cell death, including apoptosis, necroptosis, pyroptosis, ferroptosis and autophagy‐dependent cell death.^1^ The high number of cell death forms can confuse but also inspire researchers to explore these mysteries. Publications on this subject have rapidly increased since the 1990s. However, most of the mechanisms underlying cell death are still veiled. Understanding the meaning and consequence of cell death, especially the “active” forms, are difficult, similar to the riddle raised by Douglas R. Green on this topic: How dispensable is something that is essential?^2^ Perhaps, as Douglas R. Green reminds us, we should look for answers in the consequences of cell death for the remaining living cells in the organism.^3^

Ferroptosis is an iron‐dependent, non‐apoptotic RCD process named by Scott J. Dixon in 2012. Small molecules, such as erastin and RSL3, can trigger ferroptosis, which is distinct from apoptosis, necrosis and autophagy‐dependent cell death in morphology, biochemistry and gene expression.^4^ Recently, ferroptosis has become a hot topic in a variety of diseases, especially cancer therapy.^5^, ^6^ For instance, new findings reveal that cell density can affect the sensitivity to ferroptosis, and another study showed that ferroptosis can spread through cell populations in a wave‐like manner.^7^, ^8^ These factors should be considered when ferroptosis is applied to cancer therapy. In addition, some groups have tried to use nanoparticles and exosomes as carriers of erastin and drugs to precisely induce ferroptosis in tumour tissues.^9^, ^10^ These new findings and treatment attempts enrich the study of ferroptosis. Therefore, it is meaningful to review the main mechanisms underlying ferroptosis and their potential treatment value.

## 2 A PREQUEL TO FERROPTOSIS

The cognition of ferroptosis is a cumulative process. Before Dixon defined ferroptosis, the key molecules associated with it had been reported. For example, the cystine and glutamate transport system (System X_c‐_) was discovered in 1986, and scholars found that exposure to high levels of glutamate or low levels of cysteine could cause a decrease in glutathione and accumulation of intracellular peroxides.^11^, ^12^ Further, Dolma team used synthetic, lethal, high‐throughput screening to filtrate a mass of compounds for their potency to kill RAS‐mutated tumour cells and found one chemical compound, erastin, that could cause the death of cancer cells in a non‐apoptosis manner.^13^ Five years later, another two small molecules, named RSL3 and RSL5, were identified and found to lead to the death of RAS‐mutated cancer cells in an iron‐dependent, non‐apoptotic cell death manner.^14^ At the same time, a new finding emerged that GPx4 depletion caused tremendous lipid peroxidation and cell death with an unrecognized cell death pattern, which was 12/15‐lipoxygenase‐dependent and AIF‐mediated.^15^ Based on these studies, the Scott J. Dixon and team expanded, extended and systemically summarized this special type of cell death, naming it ferroptosis, which is a type of RCD caused by iron‐dependent lipid peroxides and shares none of the characteristic morphologic features associated with necrosis, apoptosis or autophagy‐dependent cell death.

## 3 MAIN MECHANISMS OF FERROPTOSIS

### 3.1 The role of lipid peroxides in ferroptosis

The most prominent feature of ferroptosis is iron‐dependent lipid peroxides. Lipid peroxides are generally viewed as eventual executioners of ferroptosis through their ability to cause plasma membrane damage.^16^ Physiologically, most intracellular oxygen is reduced to H_2_O via oxidative phosphorylation in the mitochondrial inner membrane.^17^ However, a small proportion of oxygen will participate in other physiological or biochemical activities, including phagocytosis, immune activation and xenobiotic metabolism, and result in harmful intermediates, such as reactive oxygen species (ROS).^18^, ^19^ Objectively speaking, a low and controlled ROS level is crucial for normal cellular and organismal function, and a moderately increased ROS level is beneficial for cancer development, but high ROS levels will cause cell injury and death.^20^

Current researches studied that accumulation of polyunsaturated fatty acid (PUFA) oxides is a hallmark of ferroptosis, but the accumulation process is complicated. The process involves inhibition of deoxidation, which mainly refers to glutathione peroxidase 4 (GPx4) and ferroptosis suppressor protein 1 (FSP1), and enhancement of hyperoxidation, which is catalysed by iron and a series of enzymes. Eventually, PUFA oxidation accumulates and leads to membrane integrity damage and ferroptosis.^19^, ^21^, ^22^, ^23^, ^24^ Lipoxygenases (LOXs) are the most important lipid oxidation enzymes for ferroptosis. LOX family members are iron‐incorporating enzymes that can catalyse the dioxygenation of PUFAs.^25^ Moreover, erastin‐induced cell death can be prevented if arachidonate lipoxygenase (ALOX) is silenced, and several LOX inhibitors can also have this effect.^26^, ^27^, ^28^

**Figure 1:**
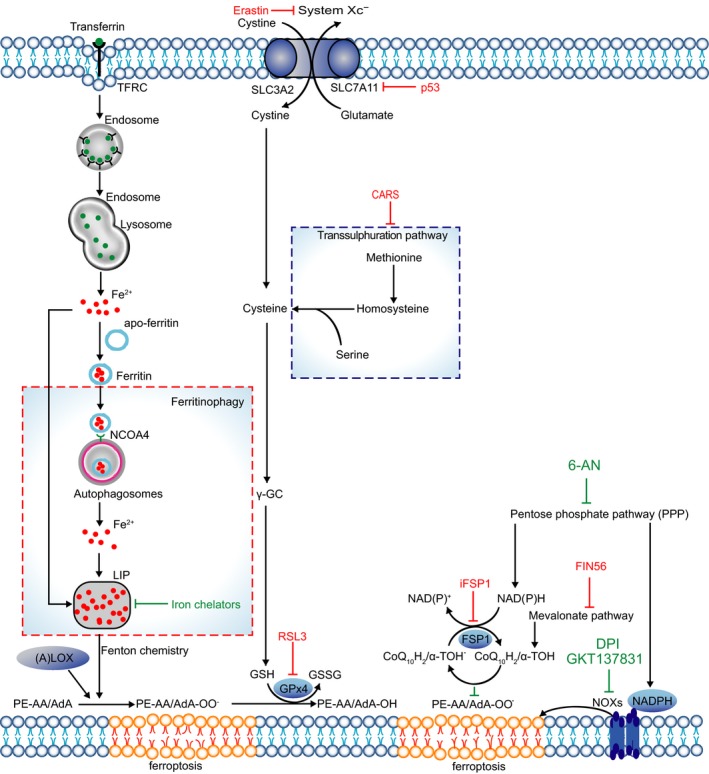
Main mechanisms of ferroptosis. TFRC‐mediated endocytosis promotes the uptake of transferrin. Ferritin can be degraded by autophagy through ferritinophagy. The LOXs, NOXs and increased labile iron result in lipid peroxide. Cysteine can be generated from uptake of cystine via system Xc^‐^ or transsulphuration pathway. GPx4 catalyses reduction reaction at the cost of GSH. CoQ10H2/α‐TOH traps lipid peroxyl radicals and FSP1 catalyses the regeneration of CoQ10

In addition, given that mitochondria are main site of redox reactions, it is rational to speculate that toxic ROS is generated by this organelle. However, Dixon's experiments found that mitochondria were not the source of the fatal ROS levels in erastin‐treated cells.^4^ To find the potential source of lethal ROS, they tested the role of the NADPH oxidase (NOX) family (NOX1–5, DUOX1, 2) in erastin‐induced ferroptosis. The results demonstrated that diphenylene iodonium (DPI, an inhibitor of canonical NOX), GKT137831 (a specific inhibitor of NOX1/4), and 6‐aminonicotinamde (6‐AN, an inhibitor of the NADPH‐generating pentose phosphate pathway) could strongly suppress erastin‐induced ferroptosis in Calu‐1 cells, a cell line that express a high level of NOX1 with RAS mutation.^4^, ^29^, ^30^ These results revealed the other source of lethal ROS, but this phenomenon was limited to several cell lines (Figure 1).^3^

Molecular dynamics showed that increased lipid peroxidation directly enhances membrane permeability, changes membrane shape and curvature, and promotes increased accessibility to oxidants, eventually causing cell death.^31^, ^32^, ^33^ A recent study demonstrated that erastin and lipid peroxidation could activate the ASK1‐p38 pathway and trigger ASK1‐dependent cell death in a cell type‐specific manner. Further results indicated ASK1 was a downstream of lipid peroxidation in ferroptosis. However, these findings only apply in special cases.^34^

### 3.2 The role of glutathione in ferroptosis

System Xc^−^ is important for the exchange of glutamate and cystine at a ratio of 1:1 across the plasma membrane.^11^ As early as 1989, it was reported that inhibition of system Xc^−^ could increase oxidative stress and mediate the toxicity of glutamate.^35^ Subsequently, researchers discovered that small molecule compounds, erastin or other system Xc^−^ inhibitors, could induce ferroptosis.^4^, ^13^, ^36^ Indeed, cystine, imported into the cytoplasm, will be catalysed to cysteine and then to GSH (glutathione) via a multistep enzyme‐catalysed reaction. GSH is an important substrate for GPx4 to reduce phospholipid hydroperoxide (PLOOH).^37^ Of note, another cysteine biosynthesis pathway, called the transsulphuration pathway, exists and generates cysteine via transfer of the sulphur atom of methionine to serine, and this pathway endows some cells with the ability to resist ferroptosis induced by system Xc^−^ inhibitors. Further study revealed that knock‐down of CARS (cysteinyl‐tRNA synthetase) upregulated the transsulphuration pathway by causing cystathionine accumulation and upregulating genes associated with serine biosynthesis and transsulphuration, thereby antagonizing erastin‐induced ferroptosis (Figure 1).^38^

GPx4 is the articulation point of ferroptosis‐related glutathione metabolism and lipid peroxidation. GPx4 determines the fate of cells because it can directly reduce the lethal phospholipid hydroperoxides.^39^, ^40^ Ras‐selective lethal (RSL) small molecules, including RSL3 and erastin, can directly decrease the activity of GPx4 by binding to it or indirectly inhibiting the import of cystine, respectively, thus leading to ferroptosis. Interestingly, GPx4 can be classified into three types according to organelle location, mGPx4 (transported into mitochondria), nGPx4 (localized in nucleoli) and cGPx4 (localized in the cytosol and nucleus), and the three GPx4 types independently regulate local lipid hydroperoxides.^40^, ^41^, ^42^, ^43^ Mitochondrial GPx4 is associated with apoptosis by inhibiting the release of cytochrome c from mitochondria.^40^, ^44^, ^45^, ^46^ While cGPx4 is associated with ferroptosis. Importantly, the Dixon team found that cells with depleted mitochondrial DNA could still trigger potent ferroptosis.^4^ The organelle‐specific GPx4 in cells might provide an explanation for why mitochondria are dispensable for ferroptosis. However, a recent study revealed that mitochondria are involved in cysteine deprivation‐induced ferroptosis via the electron transfer chain (ETC) or TCA cycle. In addition, inhibition of glutaminolysis could lead to a parallel prohibitive effect. These findings indicate that mitochondria play a decisive role in cysteine deprivation‐induced ferroptosis but not in GPx4 inhibition‐induced ferroptosis.^47^

### 3.3 Iron metabolism in ferroptosis

Intracellular iron is under delicate regulation to sustain iron homoeostasis. Iron regulatory proteins (IRP1 and IRP2) modulate the cellular Fe^2+^ concentrations, and various proteins regulate iron import, storage, release and export.^48^ Most intracellular Fe^2+^ is stored in ferritin and iron‐containing proteins, and the amount of free Fe^2+^, also called the cellular labile iron pool (LIP), is very limited.^31^ In mammalian cells, a portion of the cellular iron can be distributed in mitochondria, the cytosol, the nucleus and lysosomes; although the amount is little, cells are sensitive to iron concentration, and a little fluctuation in concentration can cause a great response.^49^, ^50^

How to increase the level of labile iron in cells? First, uptake more iron from the extracellular environment. Dixon identified six high‐confidence genes in erastin‐induced ferroptosis, and IREB2 (iron response element binding protein 2) was one of them. If IREB2 was silenced, the expression of iron uptake, metabolism and storage genes, such as TFRC, ISCU, FTH1, and FTL, would decrease, and the cells exhibited resistance to erastin‐induced ferroptosis.^4^, ^51^, ^52^ Transferrin is an iron carrier protein in serum that can be transported into cells through TFRC‐mediated endocytosis. Silencing of TFRC significantly inhibited conditional serum‐induced ferroptosis.^36^ In other words, transferrin import is required for ferroptosis. The second way to release iron is autophagic degradation of ferritin, which is called ferritinophagy.^53^ This process is primarily associated with autophagy. A recent study found that silencing of Atg5 and Atg7 (autophagy‐related genes 5 and 7) decreased intracellular Fe^2+^ levels and lipid peroxidation and further limited erastin‐induced ferroptosis. Further study confirmed that during autophagy ferritin is degraded to release labile iron and the degradation process is regulated by NCOA4 (nuclear receptor coactivator 4), a selective cargo receptor for specific autophagy of ferritin. Blockage of autophagy or genetic inhibition of NCOA4 can inhibit ferritin degradation and suppress ferroptosis.^54^, ^55^, ^56^

How does increased labile iron result in lipid ROS? Indeed, it primarily depends on Fenton chemistry and LOXs. Fenton chemistry also refers to the Fenton reaction. For this reaction, there must be enough labile iron in this cycle. The increased labile iron portion (LIP) in ferroptosis exactly meets this condition. In this reaction, peroxides and Fe^2+^ are used to produce oxygen‐centred radicals.^57^ This was further confirmed by results showing that antioxidants could terminate the reaction.^58^ If iron chelators, such as deferoxamine (DFO), were applied to downregulate intracellular iron, then iron‐dependent lipid peroxidation formation is also restrained.^4^ The indirect way for iron to generate lipid ROS is through the catalytic activity of iron‐containing enzymes, and LOXs are the most important enzymes among these (Figure 1).^59^

### 3.4 FSP1‐NAD(P)H pathway in ferroptosis

Although GPx4 plays a crucial role in ferroptosis, certain cancer cell lines are resistant to ferroptosis caused by GPx4 inhibitors,^60^, ^61^ indicating that there might be additional factors that regulate ferroptosis. To unveil the underlying mechanism, Kirill Bersuker used a synthetic lethal CRISPR–Cas9 screen to distinguish genes in U‐2 OS osteosarcoma cells treated with a GPX4 inhibitor,^62^ and Sebastian Doll generated a cDNA expression library derived from an MCF7 ferroptosis‐resistant cell line and screened for genes complementing loss of GPX4.^63^ Coincidentally, they simultaneously found that ferroptosis suppressor protein 1(FSP1) is an important ferroptosis suppressor that parallels GPx4. In FSP1‐NAD(P)H pathway, coenzyme Q10 (CoQ10) can directly trap lipid peroxyl radicals to reduce lipid peroxides, FSP1 catalyses the regeneration of CoQ10 at the cost of NAD(P)H. Sebastian Doll also screened approximately 10,000 compounds to identify FSP1 inhibitors. iFSP1 was identified as a potent FSP1 inhibitor and can trigger ferroptosis in GPX4 knockout cells that overexpress FSP1 (Figure 1).^63^

## 4 ROLE OF KRAS IN FERROPTOSIS

RAS proteins play a causal role in human cancer, and the widespread prevalence of RAS mutations (KRAS, NRAS, HRAS) in human cancer has been recognized for many years. That mutations in KRAS alone account for approximately one million deaths per year worldwide have inspired multiple attempts to find KRAS inhibitors.^13^, ^64^ These attempts have led to the discovery of RSL. Ferroptosis was coined in this background. RAS‐mutant cancer cells are sensitive to erastin or RSL3‐induced ferroptosis. However, the underlying mechanism is unclear.

**Figure 2:**
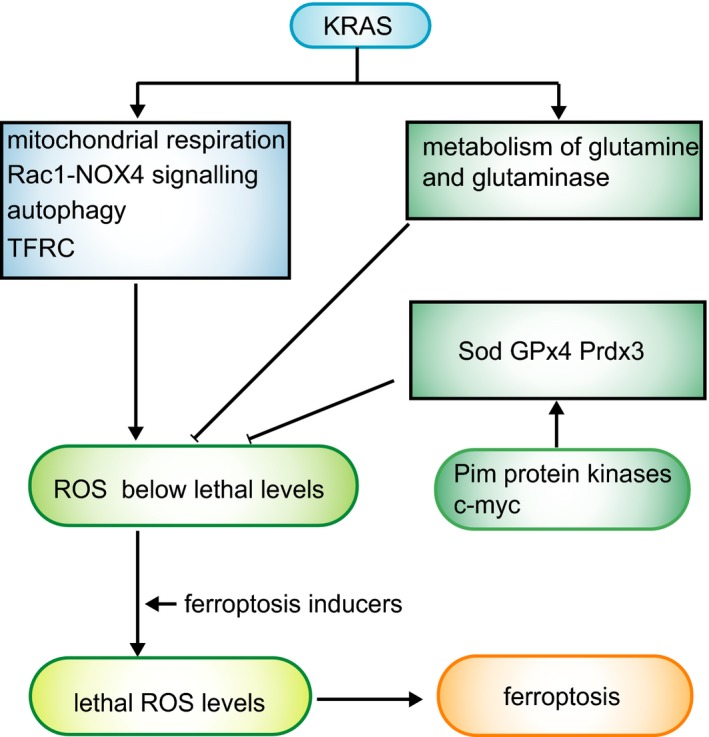
Roles of KRAS and p53 in ferroptosis. KRAS upregulates ROS by mitochondrial respiration, Rac1‐NOX4 signalling, autophagy and TFRC. Meanwhile, KRAS downregulates ROS by mediating metabolism of glutamine and glutaminase. In addition, Sod GPx4 Prdx3 help to eliminate ROS caused by KRAS. Ultimately, ROS is maintained at a moderate high level that below lethal levels. In this case, if cells were treated with ferroptosis inducers, this balance would be disrupted, thus leading to lethal ROS levels and cell death

Indeed, oncogenic KRAS can regulate metabolic changes and alter cellular signalling, both of which can increase the production of intracellular reactive oxygen species (ROS), but at the same time, it also upregulates antioxidant systems to balance ROS to levels at which they are beneficial for tumour development and progression, while remaining below the threshold that causes cell death.^65^ KRAS can upregulate ROS through multiple mechanisms. For example, KRAS can regulate HIF‐1α and HIF‐2α target genes to modulate mitochondrial metabolism or regulate the transferrin receptor (TFRC) to modulate mitochondrial respiration and ROS generation.^66^, ^67^ Notably, TFRC is a regulator in ferroptosis, as mentioned above. KRAS can also activate Rac1‐NOX4 signalling to alter NADPH oxidase activities.^68^, ^69^ The NOX family also plays an important role in lipid peroxidation in ferroptosis. In addition, KRAS controls the regeneration of peroxiredoxins by inducing autophagy‐specific genes 5 and 7 (ATG5, ATG7) and repressing SESN3.^70^, ^71^ Autophagy is also important in ferroptosis. With regard to antioxidant systems to sustain redox homoeostasis in the presence of KRAS‐induced ROS production, a recent study showed that superoxide dismutase (Sod), glutathione peroxidase 4 (GPx4) and peroxiredoxin 3 (Prdx3) are indispensable to mitigate RAS‐induced ROS. Pim protein kinases are involved in multiple cellular processes, such as signal transduction, cell cycle progression, cell metabolism and tumour growth. 72, 73, 74 However, a recent study demonstrated that all three isoforms of Pim protein kinases knockout (TKO) reduced the expression of Sod, GPx4 and Prdx3, thus leading to mouse embryo fibroblasts (MEFs) becoming susceptible to killing by K‐Ras^G12V^‐mediated ROS production.^72^ In contrast, transduction of the TKO cells with c‐Myc permitted KRAS‐induced cell growth by decreasing RAS‐induced ROS accumulation.^72^ In addition, KRAS also regulates cellular metabolism, especially the metabolism of glutamine and glutaminase,^75^, ^76^ which are associated with ferroptosis. In conclusion, small molecules that target system Xc^−^, GPx4 and autophagy disrupt the redox homoeostasis in RAS‐mutant cancer cells and eventually cause cell death in a manner referred to as ferroptosis. However, as a counterexample, overexpression of RAS (KRAS, HRAS, NRAS) in RMS13 rhabdomyosarcoma cells can protect cells from oxidative stress‐induced ferroptosis.^77^ Perhaps the effects of the RAF‐MEK‐ERK pathway on ferroptosis vary among cell lineages or with RAS‐mutant protein expression levels.^78^ This reminds us to consider genetic background in application of ferroptosis to treat cancer (Figure 2).

## 5 ROLE OF P53 IN FERROPTOSIS

p53 is a crucial tumour suppressor that orchestrates specific cellular responses, such as transient cell cycle arrest, cellular senescence and apoptosis.^79^ A recent study revealed that p53 is also involved in regulation of ferroptosis. The Le Jiang and colleagues generated a tetracycline‐controlled (tet‐on) p53‐inducible cell line for microarray analysis to identify novel p53 target genes, and SLC7A11 (a component of system Xc^−^) was identified as a novel p53 target gene. p53 could transcriptionally suppress SLC7A11 and thereby inhibit cystine uptake and sensitize H1299, U2OS and MCF7 cells to ferroptosis. In addition, p53^3KR^, an acetylation‐defective mutant that failed to induce cell cycle arrest, senescence and apoptosis, retained the ability to inhibit SLC7A11 expression and thereby downregulated cystine metabolism and ferroptosis upon ROS‐induced stress.^80^ Another study reported that p53 repressed the expression of SLC7A11 during erastin treatment by decreasing H2Bub1 occupancy on the SLC7A11 gene regulatory region.^81^ However, p53 regulation of SLC7A11 was cell line dependent. DPP4 (dipeptidyl‐peptidase‐4) can bind to NOX1, leading to lipid ROS and subsequent ferroptosis in p53‐deficient human colorectal cancer (CRC) cells. However, in p53 wild‐type cells, p53 induces DPP4 localization to the nucleus where DPP4 binds p53 to promote the expression of SLC7A11, which inhibits erastin‐induced ferroptosis, indicating that p53 has a pro‐survival function.^82^ Wild‐type p53 has been reported to delay the onset of ferroptosis in response to cystine deprivation via the p53‐p21 axis to mitigate the depletion of intracellular glutathione and reduce accumulation of lethal lipid ROS.^83^ In addition, certain mutant forms of p53 do not suppress the expression of SLC7A11. For example, a p53 that harboured mutations in the N‐terminal domain of the gene did not downregulate SLC7A11.^84^, ^85^

Conversely, p53 promotes ROS accumulation via regulation of cytochrome c oxidase 2 (SCO2), glucose transporter (GLUT)1, GLUT4 and glutaminase 2 (GLS2).^86^, ^87^, ^88^, ^89^ p53 can upregulate arachidonate 15‐lipoxygenase (ALOX15) via spermidine/spermine N1‐acetyltransferase 1 (SAT1), causing lipid peroxidation.^86^ Bo Chu and team performed an RNAi‐mediated loss‐of‐function screen to identify which lipoxygenases (ALOXE3, ALOX5, ALOX12, ALOX12B, ALOX15 and ALOX15B) affect p53‐dependent ferroptosis. The results demonstrated that inactivation of ALOX12 diminished p53‐mediated ferroptosis induced by ROS stress. SlC7A11 could bind to ALOX12 and thus inhibit the lipoxygenase activity of ALOX12. p53 can upregulate the enzymatic activity of ALOX12 by repressing SLC7A11 expression.^90^ In these conditions, p53 acts as a positive regulator of ferroptosis. Beyond doubt, this complicated genetic background of p53 will bring more challenge for cancer treatment.

## 6 PROGRESS IN FERROPTOSIS APPLICATION IN CANCER THERAPY

In the process of exploring how to kill RAS‐mutant cancer cells, ferroptosis was accidentally discovered. The ultimate purpose of elucidating the mechanism underlying ferroptosis is to obtain better cancer treatment options. Because, based on the molecular regulation mechanisms, we can specifically target the key regulators (such as system Xc^−^, GPx4, autophagy and iron) to trigger ferroptosis. Here, we list some new findings in the utilization of ferroptosis activators as weapons to treat cancer.

### 6.1 Death propagation

Although the forms of cell death vary in mechanism, from the perspective point of cellular population, the death of an individual cell can exert three types of effects on the population: first, a neutral effect on surrounding cells or a benefit that counteracts the harm; second, a benefit for neighbouring cells; and third, killing of neighbouring cells via a bystander effect.^8^ Apoptosis was initially identified as an active form of cell demise intended to degrade the cargos of doomed cells but that does not affect neighbouring cells (cell‐autonomous suicide); however, a recent study showed that these eliminable cells can trigger various signals that have a positive or negative influence on surrounding cells and tissues.^91^ Entosis is a process in which one cell (winner) engulfs another cell (loser); the loser is engulfed, or cannibalized while alive, and subsequently, undergoes cell death. The dead cells benefit the living cells.^92^ In contrast, ferroptosis can propagate among cells in a wave‐like manner, exhibiting a potent killing effect on neighbouring cells.^8^, ^93^, ^94^ Ultrasmall nanoparticle‐induced ferroptosis can cause rehabilitation of xenograft tumours, demonstrating that ferroptosis has potent tumour suppressive ability.^93^ The underlying mechanism of death propagation is unclear; however, the following factors might be involved. Large regions of dead cells that form in the interior of cancer lesions might increase the inner pressure that restricts key nutrients diffused from blood vessels and lead to energy deprivation. In this condition, necrosis of vast cell populations may occur secondary to a small portion of apoptotic cell death in the interior.^8^, ^95^, ^96^, ^97^ However, whether ferroptosis induces the demise of surrounding cells in this manner is unclear. Intriguingly, there some collective features shared by radiation and ferroptosis; for instance, they both destroy cells through lipid peroxidation that is regulated by glutathione.^98^, ^99^ Moreover, they both promote the expression of cyclooxygenase‐2 (COX‐2), and COX‐2 plays a vital role in the radiation‐induced bystander effect.^8^, ^100^, ^101^ Therefore, is there a mechanism in ferroptosis similar to the radiation‐induced bystander effect?

**Figure 3:**
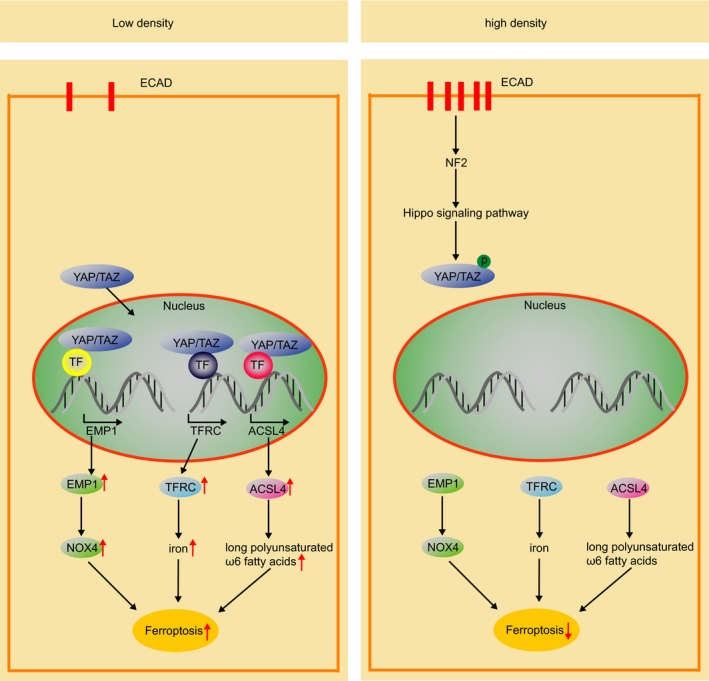
Hippo signalling pathway. Left, cells grow in low density; the Hippo signalling pathway is closed. YAP/TAZ enter nucleus to promote expression of EMP1, TFRC and ACSL4, thus cells are sensitive to ferroptosis. Right, cells grow in high density; the Hippo signalling pathway is activated. Phosphorylated YAP/TAZ is impeded in cytoplasm, thus cannot promote the expression of EMP1, TFRC and ACSL4 and cells exhibited resistance to ferroptosis

However, although we are glad that the propagation of cell death may contribute to cancer therapy, another study reported that a high cell density (>80% confluency) makes cells less sensitive to erastin‐induced ferroptosis via the Hippo pathway that regulates TAZ‐EMP1‐NOX4 axis (Figure 3).^7^ In addition, recent research has demonstrated that cell–cell contacts inhibit erastin‐induced ferroptosis and t‐BuOOH‐induced ferroptosis by decreasing basal and lipid peroxidation. Contact between cells can also antagonize ROS‐induced cell death by inhibiting the dissipation of MMPs and the increase in DNA DSBs.^102^ These findings seem to be incompatible with death propagation, but the underlying explanation for these phenomena may lie in the differences between in vivo and in vitro conditions.

### 6.2 Molecular carrier

Although RSL has been studied for many years, they are not suited for direct use in clinical applications due to their poor water solubility, renal toxicity and other toxic side effects. Thus, enhancing the specificity and delivery efficiency of these drugs is a promising research direction. Recently, exosomes and nanosystems have been found to function well in this aspect.

Exosomes are nano‐sized extracellular vesicles (40‐150 nm) with a membrane lipid bilayer that are released by all living cells and efficiently enter other cells to transfer substances and signalling information.^103^, ^104^ Recently, a great number of studies have found that exosomes have better advantages in drug delivery due to their low immunogenicity, high biocompatibility and high efficiency.^9^ One team constructed an erastin‐loaded exosome formulation labelled with folate (FA) (erastin@FA‐exo) to target triple‐negative breast cancer (TNBC) cells with folate receptor overexpression. Compared with erastin@exo and free erastin, erastin@FA‐exo increased the uptake efficiency of erastin in MDA‐MB‐231 cells and promoted ferroptosis by inhibiting system Xc^‐^.^9^

In oestrogen receptor‐positive (ER^+^) breast carcinoma, mutation of the PI(3)K/Akt signalling pathway is frequent and causes excess intracellular glutathione (GSH) biosynthesis, leading to oxidative stress resistance and activation of tumour cell proliferation and infiltration.^105^ Researchers have developed a nanosystem called drug‐organics‐inorganics self‐assembled nanosystem (DFTA). In this nanosystem, doxorubicin (DOX) was used as a chemotherapeutic agent, ferric chloride (FeCl_3_) as a ferroptosis inducer and tannic acid (TA) as an activator of a superoxide dismutase (SOD)‐like reaction in an intracellular cascade. Photothermal excitation efficiently triggered drug release from the DFTA nanosystem. The results showed that combined use of the DFTA nanosystem and laser excitation significantly reduced the level of intracellular GSH through a series of reactions. This effect was further confirmed by evaluation of the antitumour efficiency in vivo, which revealed that the tumour inhibition ratio of in the DFTA nanosystem + laser group was as high as 93.38%, even though the dose of iron and DOX was reduced by approximately 9 and 1.5 times, respectively. In a word, the nanosystem based on triple combination therapy with chemotherapy, ferroptosis and PT greatly improved the treatment specificity, reduced the toxicity and enhanced the delivery efficiency.^106^

One study reported that ultrasmall (<10 nm in diameter) silica nanoparticles can trigger ferroptosis in starved cancer cells and cancer‐bearing mice. This nanosystem was called αMSH‐PEG‐C' dots and was found to be imported into lysosomes. The surface of this nanosystem was functionalized with αMSH (alpha‐melanocyte stimulating hormone), which recognizes a special surface receptor expressed on malignant melanoma cells (melanocortin‐1 receptor, MC1‐R), thereby orienting this system specifically to malignant melanoma cells. Treatment of amino acid‐starved cells with high concentrations of these nanoparticles induced ferroptosis, and the cell death propagated in a wave‐like manner.^93^

Another nanosystem called nanoparticle ferritin‐bound erastin and rapamycin (NFER) is an Abraxane‐inspired nanodrug. When NFER is engulfed by cancer cells, the nanosystem can release erastin and rapamycin, inhibiting system Xc^‐^ and autophagy, respectively, and eventually causing cell death via ferroptosis.^10^ In addition, a metal−organic network (MON) encapsulating p53 plasmid (MON‐p53) and combined with ferric ions can also induce ferroptosis by releasing iron and p53 plasmid to inhibit system Xc^‐^. In this study, MON‐p53 triggered a “bystander effect” to sensitize cancer cells to MON‐p53‐induced ferroptosis. In tumour‐bearing mice, MON‐p53 treatment both suppressed tumour growth and prolonged the mouse life span.^107^

To specifically release iron to tumour tissues, one team developed a nanoprobe that consisting of upconversion luminescence (UCL) nanoparticles as a core and a coordinated Fe^3+^/gallic acid complex as a shell. This nanoprobe can release Fe^3+^ only in tumour lesions in response to the lightly acidic tumour pH and thus triggered ferroptosis.^108^ Magnetosomes can also be iron carriers. Another team engineered a magnetosome with an Fe_3_O_4_ magnetic nanocluster (NC) as the core and pre‐engineered leucocyte membranes as the cloak. In addition, TGF‐β inhibitor (Ti) was loaded inside the membrane and PD‐1 antibody (Pa) was anchored on the membrane surface. In this way, the magnetosome induced ferroptosis and regulated immunomodulation to treat cancer (Table 1).^109^

**Table 1:** List of carriers

Carrier	Molecule	Target	Other components	Cell lines	Ref
Exosome	Erastin	System Xc^‐^	Folate (FA)	Triple‐negative breast cancer cells	9
DFTA	FeCl_3_	LIP	DOX TA	ER^+^.breast carcinoma	106
NFER	Erastin rapamycin	System Xc^‐^ autophagy	‐	Mouse breast cancer cell line	10
MON	Ferric ions p53‐plasmid	LIP SLC7A11	‐	HT1080	107
UCNP	Fe^3+^	LIP	Gallic acid	LS180 cells	108
Magnetosome	Fe_3_O_4_	LIP	TGF‐β inhibitor PD‐1 antibody	B16F10	109
Sal–AuNPs	Salinomycin	Mitochondrial	‐	Breast cancer stem cells (BCSCs)	124
CaP‐RSL3	Fe^3+^ RSL3	LIP GPx4	Ascorbate (Asc)	Murine.breast cancer cells (4 T1)	134
Ce6‐erastin	Erastin	System Xc^‐^	Chlorin e6 (Ce6)	OTSCC	135

### 6.3 Cancer stem cells

Cancer stem cells (CSCs) have self‐renewal and infinite proliferation capability and are vital to sustaining cancer proliferation, drug resistance, metastasis and recurrence. Cancer stem cells are also important therapeutic targets.^110^ The stemness of CSCs can be verified by detecting the expression of typical surface markers (CD133, CD44, CD24, CD38) and pluripotency factors (Sox2, Oct4, Nanog) and by their capacity to form spheres. William's team found a new compound that can inhibit system Xc^‐^ to induce ferroptosis in cancer stem cells.^111^ Different from general cancer cells, increased iron content in CSCs is a key feature in several types of tumours. Correspondingly, iron chelation can inhibit the stemness of CSCs, whereas iron supplementation can have the reverse effect. In other words, iron may function as a protective factor in CSCs.^112^, ^113^, ^114^, ^115^, ^116^, ^117^ However, iron deprivation has also been found to halt cell proliferation in mouse‐induced pluripotent cells and inhibit the expression of stemness markers.^117^ These findings indicate that iron may have a dual function in CSCs. Studies have shown that levels of both the iron storage protein ferritin and the iron exporter ferroportin (FPN) are decreased in cholangiocarcinoma CSCs and ovarian CSCs.^112^, ^118^ In addition, low H ferritin levels and high TFRC expression were detected in cholangiocarcinoma cells when grown in monolayers; however, when the same cell lines formed tumour spheres, there were high H ferritin levels and low TFR1 expression levels. This indicates that the labile iron that is generated from the degradation of H ferritin or mediated by TFRC is involved in the capacity to form spheres.^118^ H ferritin knock‐down inhibited glioblastoma CSC growth and impaired a stem‐like phenotype in breast cancer cells.^115^, ^119^ These studies showed that CSCs are iron‐rich and iron‐dependent. Indeed, sphere‐forming cholangiocarcinoma cells, despite higher levels of ROS and iron, show less susceptible to erastin‐induced ferroptosis than their counterparts grown in a monolayer. However, ovarian CSCs exhibited higher sensitivity to ferroptosis than non‐tumorigenic ovarian stem cells.^118^ These phenomena might be explained by the dual role of ferritin: in slow‐growing CSCs, iron is not required for proliferation and can be stored in ferritin, thereby preventing the generation of lipid peroxidation and explaining the resistance of cholangiocarcinoma cells with high ferritin content to erastin. Correspondingly, if ferritin degradation is initiated, ferritin may also represent a source of iron, thus causing ferroptosis.^117^

Salinomycin (Sal) is a polyether ionophore antibiotic that has been shown to kill CSCs in different types of human cancers.^120^ On the other hand, gold nanoparticles (AuNPs) are excellent drug carriers with good biocompatibility, easy synthesis and good drug functionalization capability.^121^, ^122^, ^123^ Studies have found that salinomycin‐loaded gold nanoparticles can induce ferroptosis in CSCs.^124^

### 6.4 Tumour microenvironment

The microenvironment is one of the most important features of tumour and plays a critical role in many aspects of tumorigenesis. Currently, the exploration of ferroptosis is primarily focused on vitro cell lines or xenogeneic tumour cell transplantation. Thus, the role of the tumour microenvironment in ferroptosis is being neglected. Iron is the key factor in ferroptosis. An aberrantly increased labile iron portion can originate from two main sources as mentioned above: import of transferrin and degradation of ferritin via ferritinophagy. TFR mediates the import of transferrin from the extracellular environment, while FPN1 is the only ferrous iron exporter. FPN1 is primarily expressed in iron‐recycling macrophage populations.^125^, ^126^ In the extracellular milieu, ferric iron is bound by apo‐transferrin (TF), which is the key iron transport protein in plasma. Hepcidin is an iron regulatory hormone that acts in a negative feedback manner through binding to FPN1.^127^, ^128^ Macrophages play an important role in sustaining iron homoeostasis. Although the mechanism of iron homoeostasis in the extracellular environment is clear, the changes in response to ferroptosis are unclear and whether these changes affect the efficiency of therapy remains to be unveiled.

In solid tumours, hypoxia and acidity characterize the microenvironment of cancer cells. Numerous iron metabolism‐associated genes, including iron transport (TFRC and SLC11A2) and iron storage (FTH, encoding ferritin heavy chain) genes, are under the control by hypoxia‐responsive elements (HRE).^129^, ^130^, ^131^ In addition, a recent study showed that hypoxia can protect cells from ferroptosis by upregulating the expression of carbonic anhydrase 9 (CA9). Carbonic anhydrases (CAs) represent a superfamily of metalloenzymes that equilibrate the reactions among CO_2_, bicarbonate and H^+^.^132^ Under hypoxia conditions, increased CA9 can enhance the formation of FTL and FTH, leading to a corresponding decrease in the protein levels of TFRC and FPN‐1 and in the labile iron portion, thus inhibiting iron‐dependent lipid peroxidation.^133^

However, despite the protective function against ferroptosis, two new therapy strategies target the acidic and hypoxic environment in tumour tissues. One study reported that ascorbate (Asc) can selectively kill cancer cells via hydrogen peroxide (H_2_O_2_) accumulation only in tumour extracellular fluids. The team synergized the action of Asc with a lipid‐coated calcium phosphate (CaP) hybrid nanocarrier that can concurrently load polar Fe^3+^ and non‐polar RSL3. Interestingly, the hybrid nanocarrier showed accelerated drug release under acidic conditions (pH 5.0). In addition, this method produced significantly elevated levels of hydroxyl radicals, lipid peroxides and depleted glutathione under hypoxia, which was accompanied by strong cytotoxicity. Moreover, this system also functioned well in a tumour‐bearing xenograft mouse model.^134^ On the other hand, the non‐invasive nature of photodynamic therapy (PDT) is an increasingly important therapeutic method with spatiotemporal selectivity for the treatment of cancer and thus enables preservation of organ function in cancer patients. However, hypoxia limits the supply of O_2_ that is required for PDT. Considering that Fenton reaction in ferroptosis will produce reactive oxygen species (ROS) and sustainably supply O_2_, another team constructed a Ce6‐erastin nanoparticle system to combine ferroptosis with PDT. Their results showed that the Ce6‐erastin nanoparticles exhibited low cytotoxicity to normal tissues but led to the death of cancer cells and achieved satisfactory antitumour effects in a xenograft tumour mouse model upon irradiation.^135^

## 7 CONCLUSION AND PERSPECTIVE

Although the main mechanisms underlying ferroptosis have been revealed, there are still many uncertain factors affecting the destiny of cancer cells, such as genetic background, cell type and cell density. Moreover, the situation is more complicated if extended to cancer therapy. For instance, if the expression of cysteinyl‐tRNA synthetase (CARS) is low in cancer cells, the transsulphuration pathway would be activated due to the accumulation of cystathionine and expression of genes associated with serine biosynthesis and transsulphuration, and in this case, using erastin alone will not achieve the expected effect.^38^ In addition, cell density is a negative regulator of ferroptosis. When cells grow at high density, E‐cadherin (ECAD) expression is increased, and ferroptosis is suppressed in epithelial cells. However, cancer cells with mesenchymal or metastatic properties, including a low level of ECAD expression, are highly sensitive to ferroptosis.^136^, ^137^ Jiao Wu's team found that ECAD expression increased as cell density increased and then activated intracellular NF2 and the Hippo signalling pathway to mediate the density‐dependent resistance to ferroptosis. Regarding the mechanism, NF2 can inhibit the expression of TFRC and acyl‐CoA synthetase long‐chain family member 4 (ACSL4) by suppressing the activity of YAP, and both TFRC and ACSL4 are crucial mediators of ferroptosis. This finding has clear implications because of the cancer therapies. Because cadherin–NF2–Hippo signalling axis is frequently mutated in cancer, these mutations endow cancer cells with sensitivity to ferroptosis (Figure 3).^137^ Acyl‐CoA synthetase long‐chain family member 4 (Acsl4) could potentiate the kill effect of GPx4 inhibitor (RSL3) via enriching cellular membranes with long polyunsaturated ω6 fatty acids. Moreover, Acsl4 could predict cancer cells’ sensitivity to ferroptosis for therapeutic approach due to its preferential expression in a panel of basal‐like breast cancer cell lines.^138^ Concerning how to precisely induce ferroptosis in vivo, the key regulators of ferroptosis, which have been uncovered, provide vital therapeutic targets, such as targeting system Xc^−^, GPx4, autophagy and iron. Researchers have found a number of small molecules that can induce ferroptosis by targeting these therapeutic targets. However, these compounds are not currently suited for direct use in vivo, but further application of cutting‐edge technology may bring new options for cancer treatment based on ferroptosis.

## CONFLICT OF INTEREST

The authors have declared no conflicting interests.

## AUTHOR CONTRIBUTION

YQ conceived of the presented idea; ZY, WL and QZ wrote the manuscript; QH, ML, QS, ZZ, GF, WX and SJ searched the literature; XX and XY supervised the project.

## Data Availability

The data that support the findings of this study are available from the corresponding author upon reasonable request.
